# Comparative Study on the Effects of Four Plant Protein Sources on the Liver and Intestinal Health of Largemouth Bass, *Micropterus salmoides*

**DOI:** 10.1155/2024/6337005

**Published:** 2024-01-24

**Authors:** Shibin Yao, Wenjian Li, Chunfang Cai, Chengrui Wang, Jia Kang, Honglin Hu, Ping Wu, Xiamin Cao, Yuantu Ye

**Affiliations:** Key Laboratory of Aquatic Animal Nutrition of Jiangsu, School of Biology and Basic Medical Sciences, Soochow University, Suzhou 215123, China

## Abstract

The effects of plant protein sources (PPSs) on the health of the liver and intestine of the largemouth bass, *Micropterus salmoides*, were compared to verify the potential damaging effects of dietary fiber (DF). A diet containing 55% fish meal (FM) was used as the control. The test diets contained 25% soybean meal (SBM), rapeseed meal (RSM), cottonseed meal, or peanut meal, and the FM content was decreased to 30%. The protein and lipid contents of these five diets were balanced by casein and oil. Fish were raised for 8 weeks. The fish fed the diet containing PPS showed a trend of decreasing growth and apparent digestibility coefficients. The contents of total bile acid, lipid, and collagen in the liver were increased, and the mRNA expression levels of genes encoding inflammatory factors and enzymes involved in *de novo* fatty acid synthesis and bile acid synthesis were upregulated. Both the lipid and collagen contents in the liver were positively correlated with the DF content in the diet significantly. Morphology and histology showed reduced liver size, hepatic steatosis, and fibrosis in fish fed diets containing PPS. The lowest hepatosomatic index was observed in fish fed the SBM diet, and the most severe damage was observed in fish fed the RSM diet. No obvious histological abnormalities were observed in the hindgut. The bile acid profile in the liver could be used to distinguish the types of PPS very well by Fisher discriminant analysis. These results indicated that 25% of each of the four PPSs in the diet exceeded the tolerance range of largemouth bass and caused liver damage, which might be mediated by bile acid. DF in PPS might be an important agent contributing to liver damage.

## 1. Introduction

With the successful application of formula feed, the aquaculture production of the largemouth bass, *Micropterus salmoides*, has been rapidly increasing in China during the past decade. However, the incidence of diseases, such as fatty liver and enteritis, is also gradually increasing. Previous studies have shown that growth retardation [1], fatty liver [2], and enteritis [3, 4] could be induced in different fish species, including largemouth bass, when the diet contains a high level of plant protein sources (PPSs) [5, 6]. However, the pathogenic agent is still unclear.

PPSs are rich in dietary fiber (DF), which refers to nondigestible carbohydrates with polymerization degrees ≥3, such as nonstarch polysaccharides, resistant starch, resistant maltodextrins, fructo-oligosaccharides, and galacto-oligosaccharides [7, 8]. The optimum DF level could alleviate fatty liver [9] and improves gut health [10, 11] in humans and animals, including fish species [12]. However, studies in recent years have also suggested that the health benefits of DF need to be redefined [13], as DF could induce cholestasis, hepatic inflammation, and liver cancer, which is microbiota-dependent and transmissible [14]. In fish, it was also observed that high levels of DF induced cholestasis, hepatic steatosis and fibrosis, and enteritis [15, 16]. These studies suggested that DF in PPS might contribute to the abovementioned fatty liver and enteritis. The type and level of DF varied among different PPS, which indicates that the impact on fish health should be different.

Soybean meal (SBM), rapeseed meal (RSM), cottonseed meal (CSM), and peanut meal (PNM) are often used to replace fish meal (FM) in aquatic formula feed to cope with the shortage of FM due to their accessibility, affordability, and high protein content [17, 18]. The DF content in these four PPSs is more than 30% [19–22]. In this study, the effects of 25% of these four PPSs in the diet on the health of the liver and gut of largemouth bass were assessed. The results revealed that all four PPSs can cause different degrees of liver tissue damage accompanied by a disorder of bile acid (BA) homeostasis, with RSM being the worst. These results are useful for understanding feeding-induced health damage as well as feed optimization.

## 2. Materials and Methods

### 2.1. Diets

Except for casein, dextrin, and yttrium oxide, which were analytical reagents, all the other feedstuffs were provided by Guangdong Yuehai Feed Co., Ltd. The feed ingredients were fully ground and screened with an 80 mesh sieve, and the undersize was collected. The approximate compositions of FM, SBM, RSM, CSM, and PNM were analyzed, and the results are shown in Table 1. A diet containing 55% FM was used as the control. The test diets contained 25% SBM, RSM, CSM, and PNM, and the percentage of FM was decreased to 30%. The 25% PPS was high enough to induce adverse effects on liver health in largemouth bass according to Weng et al. [23] and our previous study when FM was less than 30%. The protein and lipid contents of the five diets were balanced by casein and oil. Each ingredient was weighed according to the formula, mixed evenly, and then pelletized into particles of 1.0 mm in diameter. After drying, the pellets were stored at −20°C. The formulas of the five diets are shown in Table 2.

### 2.2. Feeding and Fecal Collection

An 8-week growth trial was conducted at the Postgraduate Workstation of Suzhou Yangcheng Lake National Modern Agricultural Development Co., Ltd. The water used in this experiment was introduced from Yangcheng Lake, precipitated and filtered. Juvenile largemouth bass were provided by a local farm and temporarily raised in an indoor cement pond for 20 days. During this time, fish were fed commercial feed. A total of 330 healthy fish of the same size were distributed evenly among 15 tanks, with 22 fish per tank. Fish were fed the FM diet for one more week to adapt to the trial conditions. Then, in a state of fasting, all fish in each tank were netted, and two fish of proper size were picked out to allow the total weight of the remaining 20 fish to be equal among tanks (CV < 3%). The average body weight was 12.5 g at that time. Fish were then fed the five experimental diets manually in three duplicates, with a random distribution, two times a day (at 8:00 and 17:00) to apparent satiety. As DF-induced damage was microbiota-dependent and transmissible [14], water was not recycled. The sewage was siphoned at 9:00 everyday, and one-third of the bottom water was discharged. Fresh water was then immediately and gently added. Dead fish (occurring only in the first week) were weighed and substituted by a similar size healthy fish selected from the cement storage pond. The feces were collected everyday from the fourth week and stored at −20°C. During the growth trial, the water temperature was 26∼30°C, the pH was 7.5∼7.9, the DO concentration was > 6.5 mg/l, and the ammonia nitrogen concentration was <0.5 mg/l.

### 2.3. Sample Collection and Preparation

At the end of the feeding trial, fish were fasted for 24 hr, quickly netted, and anesthetized use 100 ppm MS-222. They were then weighed individually. Three fish were randomly fetched for whole body approximate composition analysis; two fish were dissected. The liver and hindgut tissues were taken and fixed with 3% paraformaldehyde for histopathological analysis. Three fish were sacrificed to sample the liver, which was frozen in liquid nitrogen, stored at −80°C for later gene expression analysis. Blood was drawn from five fish with a heparin sodium-treated syringe from the tail vein. The blood was injected into a 1.5 ml Eppendorf tube, and centrifuged at 4,000 r/min, 4°C for 10 min. Then, plasma was drawn out and stored at −80°C. These fish were further dissected to sample the liver tissue to determine the lipid, collagen, and total BA (TBA) contents. The visceral mass, liver, and gallbladder of all the above fish were weighed to calculate the viscerosomatic index (VSI), hepatosomatic index (HSI), and gallbladder somatic index (GBSI).

### 2.4. Analysis Methods

#### 2.4.1. Approximate Composition and Yttrium

The moisture in the diet was measured by drying at 105°C and in the whole fish body by a freeze-drier (LGJ-18B). The protein, lipid, and ash in the diet, feces, and whole body were determined by the GB/T 6432-2018, GB/T 6433-2006, and GB/T 6438-2007, respectively. The content of total DF and soluble DF in the diet was determined by the enzymatic–gravimetric method (GB 5009.88-2014). The content of yttrium in the feed and feces was measured with an ICP-ES (PerkinElmer Optima 8000, U.S.), before which the samples of the diet and feces were digested in AR grade HNO_3_ with a GH08Z-Z microwave digestion instrument (Gaohuan Youke, Qingdao). The approximate composition of the five diets is shown in Table 2.

#### 2.4.2. Plasma Biochemical Indices

Plasma aspartate aminotransferase (AST, aspartate substrate method), alanine aminotransferase (ALT, alanine substrate method), alkaline phosphatase (ALP, NPP Substrate-AMP Buffer Method), total cholesterol (TC, CHOO-PAP substrate method), and triglyceride (TG, GPO-PAP method) levels were determined by a biochemical analyzer (C8000, Abbott Laboratories, USA), the reagent kit was purchased from Ningbo Meikang Biotechnology Co., Ltd.

#### 2.4.3. Liver Lipid, Collagen, TBA Content, and BA Profile

The liver tissue was freeze-dried, and the lipid content was determined by the Soxhlet extraction method, as described above. The TBA content was determined also using the biochemical analyzer (Abbott C8000, U.S.) and the manufacturer of kit was Ningbo Meikang Biotechnology Co., Ltd. The collagen content was determined by a Sanjia Fish COL ELISA Kit (Suzhou, China). The BA profile was determined by the UPLC-QqQ-MS method. Briefly, frozen samples were thawed at 4°C, ground, and mixed evenly. One hundred milligrams of the sample was weighed, and 5 ml of methanol solution (1 : 1) was added. Then, it was shaken for 5 min, sonicated at 4°C for 30 min, incubated at 4°C for 30 min, and centrifuged at 12,000 rpm for 10 min. The supernatant (200 *μ*l) was collected and placed in a 1.5 ml Eppendorf tube. Then, 190 precipitants (methanol : acetonitrile = 1 : 1) were added, and 10 *μ*l methanol (including internal standard 4,000 ng/ml) was added. It was then shaken for 5 min and centrifuged at 12,000 rpm for 10 min. The supernatant was filtered through a 0.22 *μ*m PVDF membrane syringe filter for LC‒MS. Waters Acquity UPLC and AB Sciex 5500-MS were used in the study. A C-18 column (1.7 *μ*m i.d.; 2.1 mm × 100 mm) was used, and the temperature was 40°C. Mobile phase A was water with 0.05% formic acid, whereas phase B was acetonitrile with 0.05% formic acid. The flow rate was 0.45 ml/min. The gradient program used was as follows: 0–1 min, 10% B; 1–2 min, 10%–40% B; 2–5 min, 40%–45% B; 5–7.5 min, 45%–60% B; 7.5–9.5 min, 60%–65% B; 9.5–11.5 min, 65%–80% B; 11.5–13.5 min, 80% B; 13.5–14 min, 80%–10% B; and 14–16 min, 10% B. The injection volume of all samples was 6 *μ*l. All types of BA and their respective sulfates were detected in negative ionization mode with the following mass spectrometer source settings: ion source, ESI; ion spray voltage, 4500 V; source temperature, 450°C; curtain gas, 35 arb; collision gas, 9 arb; ion source gas1, 45 arb; and ion source gas 2, 45 arb. The multiple reaction monitoring transitions for each analyte and MS parameters are shown in Table 3.

#### 2.4.4. Gene Expression

The mRNA expression levels of *cyp7a1*, *cyp8b1*, *cyp27a1*, *fxr*, *nf-kb1*, *il-1β*, *tnf-α*, *il-10*, *tgf-β1*, *lxr*, *hmgcr*, *pparα*, *fas*, *acc1*, and *apob* in the liver were determined. The primer sequences are shown in Table 4. Total mRNA of liver tissue was extracted with a TRIzol kit (Takara, Suzhou, China). The mRNA concentration was examined with a nucleic acid detector (NanoDrop Lite, Thermo Fisher Scientific) after 1.0% gel electrophoresis. cDNA was synthesized with a PrimeScriptTM RT Master Mix kit, and qRT‒PCR was performed to quantify the relative expression of the target gene (CFX96 Touch, Bio-Rad, U.S.). The program was as follows: predenaturation at 95°C for 30 s; then, 40 amplification cycles at 95°C for 15 s and 60°C for 20 s. The melting cycle was 95°C for 15 s, 60°C for 30 s, and 95°C for 15 s. The amplification efficiency deviation between the target gene and the internal reference gene was less than 5%, and the 2^−*ΔΔ*Ct^ method was used to calculate the relative expression of the target gene.

#### 2.4.5. Histology

The liver and hindgut tissues fixed in paraformaldehyde were removed and washed, and then sections were made according to routine procedures. Liver tissue sections were stained with H&E, Masson, and oil red O, and intestinal tissue sections were stained with H&E. An Olympus BX51 microscope and DP72 digital imaging system were used for observation.

### 2.5. Calculation

Survival, weight gain (WG), specific growth rate (SGR), feed intake (FI), feed conversion ratio (FCR), protein efficiency ratio (PER), VSI, HSI, GBSI, and apparent digestibility coefficients (ADCs) of dietary nutrients were calculated as follows:

(1) $$\begin{array}{c} \text{Survival (\%)}=100\times \left(1-\frac{\text{Number of dead fish}\in \text{each tank}}{20}\right). \end{array}$$

(2) $$\begin{array}{c} \text{WG (\%)}=100\times \frac{\text{Final mean weight}\left(\text{g}/fish\right)\text{-initial mean weight}\left(\text{g}/fish\right)}{\text{Initial mean weight}\left(\text{g}/fish\right)}. \end{array}$$

(3) $$\begin{array}{c} \text{SGR}\left(\%\text{/day}\right)=100\times \frac{\text{ln final mean weight}\left(\text{g}/\text{fish}\right)-\text{ln initial mean weight}\left(\text{g}/\text{fish}\right)}{56\;days}. \end{array}$$

(4) $$\begin{array}{c} \text{FI}\left(\text{g/fish/day}\right)=\frac{\text{Diet intake}/\text{20 fish}}{56\;days}. \end{array}$$

(5) $$\begin{array}{c} \text{FCR}=\frac{\text{Diet intake }\left(\text{g}/\text{tank}\right)}{\text{Final body weight }\left(\text{g}/tank\right)-\text{initial body weight }\left(\text{g}/tank\right)}. \end{array}$$

(6) $$\begin{array}{c} \text{PER}=\frac{\text{Final body weight}\left(\text{g}/tank\right)-\text{initial body weight}\left(\text{g}/tank\right)}{\text{Diet intake}\left(\text{g}/tank\right)\times \text{protein content}\in diet\left(\%\right)}. \end{array}$$

(7) $$\begin{array}{c} \text{VSI (\%)}=100\times \frac{\text{Visceral weight}\left(\text{g}\right)}{\text{Body weight}\left(\text{g}\right)}. \end{array}$$

(8) $$\begin{array}{c} \text{HSI (\%)}=100\times \frac{\text{Hepatopancreas weight}\left(\text{g}\right)}{\text{Body weight}\left(\text{g}\right)}. \end{array}$$

(9) $$\begin{array}{c} \text{GBSI (\%)}=100\times \frac{\text{Gallbladder weight}\left(\text{g}\right)}{\text{Body weight}\left(\text{g}\right)}. \end{array}$$

(10) $$\begin{array}{c} \text{ADC (\%)}=100\times \left(1-\frac{\text{Nutreient content}\in \text{feaces}}{\text{Nutreient content}\in \text{diet}}\times \frac{\text{Y content}\in \text{diet}}{\text{Y content}\in \text{feaces}}\right). \end{array}$$

### 2.6. Statistical Analysis

All data were subjected to one-way ANOVA, followed by Duncan's multiple-range test. Pearson correlation was examined between DF or soluble DF and liver lipid or collagen content. Stepwise (lambda method) discriminant analysis was performed for BA profile data to screen for differential components. Statistical analysis was performed by SPSS Statistics 21.0 (SPSS Inc., USA), and the significance level was set as *P* < 0.05.

## 3. Results

### 3.1. Survival, Growth Performance, and Diet Utilization

No more than two fish died in a small number of tanks, and no fish died after the first week. The survival was higher than 96.67%, and no significant difference was detected in survival among groups (*P* > 0.05). After feeding for 8 weeks, the FBW, WG, SGR, and PER of all groups fed the diet containing PPS were lower than those of fish fed the FM diet significantly (*P* < 0.05, Table 5). The fish fed the RSM diet had the worst SGR, which was even lower than that of fish fed the PNM diet (*P* < 0.05).

### 3.2. Body Composition

There was no significant difference in the body composition of largemouth bass fed different diets (*P* < 0.05, Table 6).

### 3.3. Apparent Digestibility Coefficients

The ADC of dry matter, protein, and lipid in fish fed the diet containing PPS was lower than that fed the FM diet. The statistical analysis showed that the ADC of dry matter and protein of the RSM diet was significantly lower than that of the FM diet and PNM diet (*P* < 0.05, Table 7), and the ADC of protein of the CSM diet was also significantly lower than that of the FM diet (*P* < 0.05). No significant difference was detected in the ADC of lipids among the groups (*P* > 0.05).

### 3.4. Plasma Biochemistry

The plasma biochemical indices of largemouth bass changed significantly in fish fed a diet containing PPS compared to fish fed the FM diet, with fish fed the RSM diet showing significantly higher AST, ALT, ALP, and TG levels and lower TC levels than those fed the FM diet (Table 8). Fish fed the SBM diet, CSM diet, and PNM diet showed similar changes as fish fed the RSM diet in some of these parameters (Table 8).

### 3.5. Organ Index

The VSI and HSI in fish fed the diet containing PPS were lower than those in fish fed the FM diet (*P* < 0.05, Table 9). The fish fed the SBM diet had the lowest values, followed by those fed the RSM diet. The GBSI was increased in fish fed a diet containing PPS, and that of fish fed the RSM diet and CSM diet were significantly higher than that of fish fed the FM diet (*P* < 0.05).

### 3.6. Morphology and Histology of the Liver and Intestine

**Figure 1:**
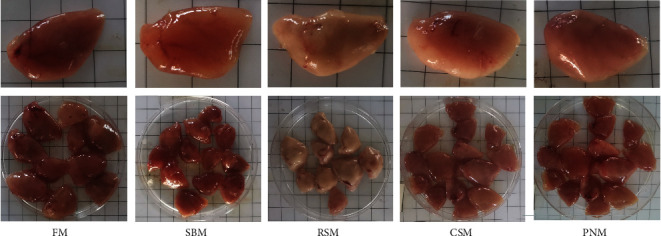
Effects of four types of plant protein sources on the size and color of the liver of largemouth bass, *M. salmoides*. The size of the square in the background is 1 cm × 1 cm.

**Figure 2:**
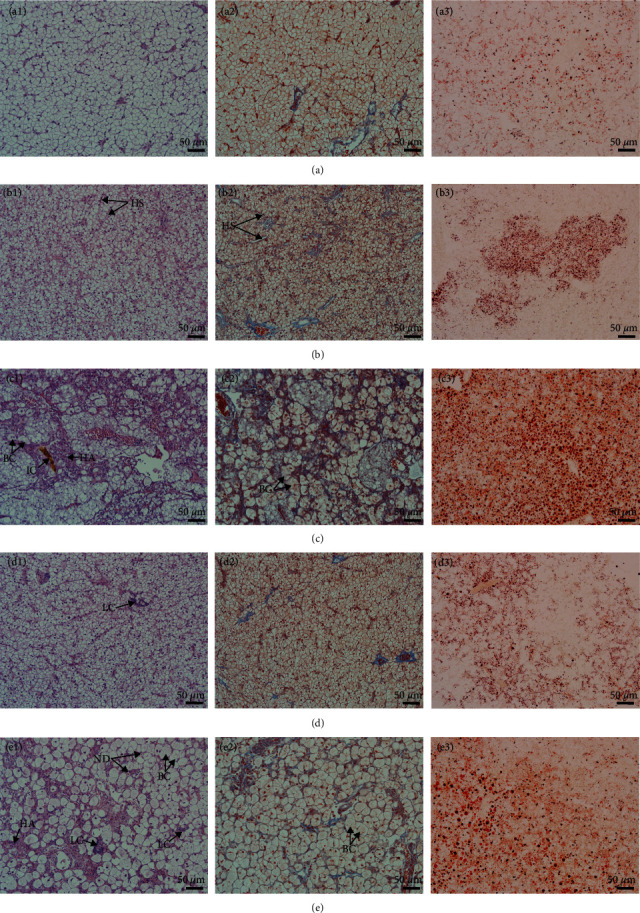
Liver tissue sections of largemouth bass, *M. salmoides*, fed FM (a(a1–a3)), SBM (b(b1–b3)), RSM (c(c1–c3)), CSM (d(d1–d3)), and PNM (e(e1–e3)) diets and stained with H&E (left column), Masson (middle column), and oil red o (right column). HS, hepatic sinus; BC, ballooning cells; HA, hepatocellular atrophy; IC, intrahepatic cholestasis; LC, lymphocyte; and ND, nuclear debris.

Figure 1 shows the liver morphology of fish fed different experimental diets. In general, the liver size trended toward a decrease in fish fed any of the diets containing PPS compared with fish fed the FM diet. Obvious differences in color were also noticed among the groups, with fish fed the RSM diet showing a white liver phenomenon. From the typical tissue section with H&E staining, the size of hepatocytes in fish fed the FM diet was uniform, and the cell boundary was clear. There was almost no blue collagen signal between hepatocytes on the Masson staining section, and the oil red O staining section showed rare and small lipid droplets (Figure 2(a1)–2(a3)). The fish fed the SBM diet showed hepatic sinusoidal dilatation and capillarization, and the number increased compared with that of the fish fed the FM diet (Figure 2(b1)). Masson-stained sections showed blue collagen signals filling the hepatocyte interstitium (Figure 2(b2)), and oil red O-stained sections showed dense lipid droplets locally (Figure 2(b3)). In fish fed the RSM diet, some hepatocytes were enlarged, and ballooning degeneration was observed. Other hepatocytes were atrophied, and the eosinophilic cytoplasm was enhanced. Diffuse steatosis and necrosis, with cholestasis, were observed (Figure 2(c1)). Masson-stained sections showed that blue collagen signals filled the liver tissue (Figure 2(c2)), and oil red O-stained sections showed that the lipid droplets were large and dense (Figure 2(c3)). In fish fed the CSM diet, local lymphocyte infiltration and a slightly increased density of hepatic sinuses were observed in H&E-stained sections (Figure 2(d1)). Enhanced blue and red signals were observed in Masson- and oil red O-stained sections, respectively (Figures 2(d2) and 2(d3)). In fish fed the PNM diet, a large number of ballooning or atrophied cells were observed in liver sections, and some liver nuclei were decomposed (Figure 2(e1)). Cholestasis could also be observed in slice. Masson-stained sections showed that the collagen signal among hepatocytes was enhanced (Figure 2(e2)), and the size of lipid droplets shown in the oil red O-stained section was enlarged (Figure 2(e3)). In general, the symptoms of liver tissue necrosis in fish fed the PNM diet were similar to those in fish fed the RSM diet, but the degree was slight.

**Figure 3:**
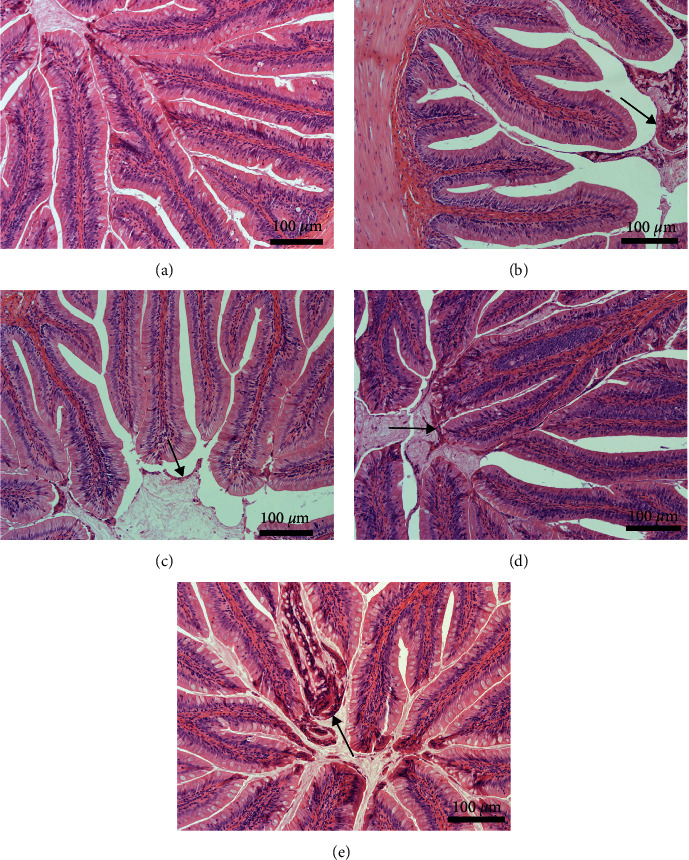
Intestinal sections of largemouth bass, *M. salmoides*, fed the FM (a), SBM (b), RSM (c), CSM (d), and PNM (e) diets, respectively, stained with H&E. Arrows indicate exfoliated epithelial cells.

There was no significant difference in the height and width of intestinal folds or the number of goblet cells among the groups (data not provided). The number of exfoliated epithelial cells in the intestinal lumen was higher in fish fed any of the diets containing PPS than in fish fed the FM diet, especially in fish fed the SBM diet and PNM diet (Figure 3).

### 3.7. Lipid, Collagen, and TBA Contents in the Liver

**Figure 4:**
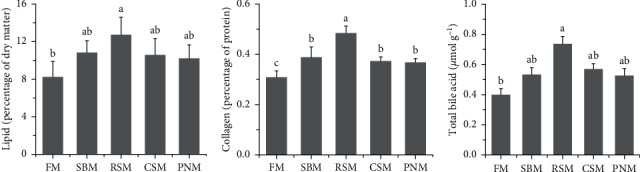
Effects of four types of plant protein sources on lipid, collagen, and total bile acid contents in the liver of largemouth bass, *M. salmoides*. The columns denoted by different letters are significantly different (*P* < 0.05, *n* = 3).

The ranking order of the groups according to lipid and collagen contents in the livers was RSM > SBM > CSM > PNM > FM, and that for the TBA content was RSM > CSM > SBM > PNM > FM (Figure 4). Statistical analysis showed that the lipid and TBA contents in fish fed the RSM diet were markedly higher than those in fish fed the FM diet (*P* < 0.05), and no significant increase was observed in fish fed the SBM, CSM, and PNM diets (*P* > 0.05). The collagen content in fish fed the RSM diet was significantly higher than that in fish fed the other diets (*P* < 0.05), and the collagen content in fish fed the SBM, CSM, and PNM diets was significantly higher than that in fish fed the FM diet (*P* < 0.05). Both lipid and collagen contents in the liver of fish fed diets containing PPS displayed a high positive correlation with total DF (*r* = 0.511, *P*=0.090; *r* = 0.743, *P*=0.006) as well as with soluble DF (*r* = 0.371, *P*=0.235; *r* = 0.535, *P*=0.073).

### 3.8. Bile Acid Composition

**Figure 5:**
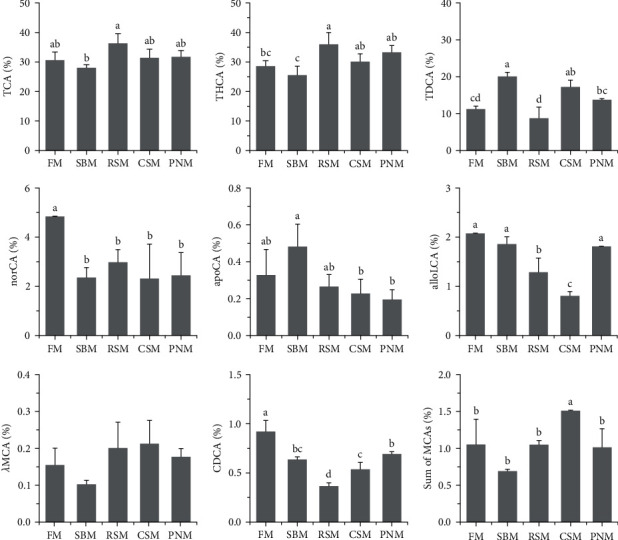
The percentage of individual bile acids in the liver of largemouth bass, *M. salmoides* fed different diets. The columns denoted by different letters are significantly different (*P* < 0.05, *n* = 3).

**Figure 6:**
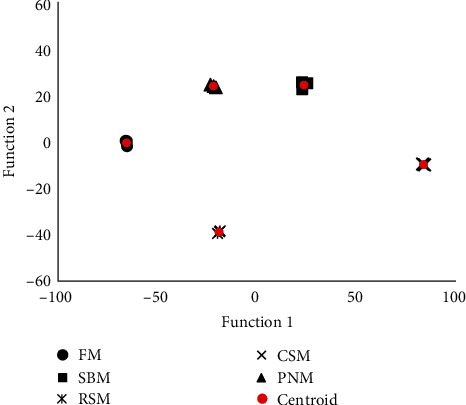
Score plot of the top two functions to discriminate diets based on the liver bile acid profile.

A total of 35 types of bile acid were detected in the liver tissue of largemouth bass (Table 3). The TCA, THCA, TDCA, and NorCA contents accounted for 30.6%, 28.6%, 11.2%, and 4.8% in all detected BAs in fish fed the FM diet (Figure 5). TDCA, apoCA, alloLCA, NorCA, THCA, and *λ*MCA were screened out and entered into the discriminative model. This model could distinguish among the diets very well (Figure 6), with the accuracy of cross-validation reaching 100%. Compared to the fish fed the FM diet, the change trends of individual BAs among fish fed the diet containing PPS were not consistent (Figure 5); however, the percentage of norCA and CDCA in fish fed the diet containing PPS decreased (*P* < 0.05), and CDCA in fish fed the RSM diet was the lowest (*P* < 0.05). The percentage of the sum of the MCAs was the highest in fish fed the CSM diet (*P* < 0.05), and no significant difference was detected among the other groups (*P* > 0.05, Figure 5).

### 3.9. Gene Expression

**Figure 7:**
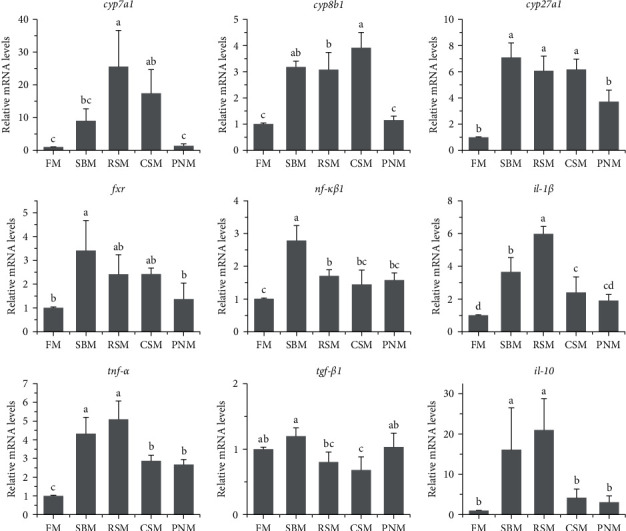
Expression levels of bile acid and immune reactivity-related genes in the liver of largemouth bass, *M. salmoides*, fed diets with different plant protein sources. The columns denoted by different letters are significantly different (*P* < 0.05, *n* = 3).

Figure 7 shows the expression of genes related to BA metabolism and inflammatory reactions in the liver. Compared with that of fish fed the FM diet, the expression of *cyp7a1* in the liver of fish fed the RSM diet and CSM diet and *cyp8b1* and *cyp27a1* in fish fed the SBM, RSM, and CSM diets was significantly upregulated (*P* < 0.05). Additionally, the expression levels of *fxr* were increased in fish fed any of the diets containing PPS; however, a significant change was detected only in fish fed the SBM diet (*P* < 0.05). The expression levels of the liver cytokine genes *nf-κβ1* and *il-10* in fish fed the SBM diet and RSM diet, those of *il-1β* in fish fed the SBM, RSM, and CSM diets, and those of *tnf-α* in fish fed the diet containing PPS were significantly increased (*P* < 0.05). The expression levels of *tgf-β1* were decreased in fish fed the RSM diet (*P* < 0.05).

**Figure 8:**
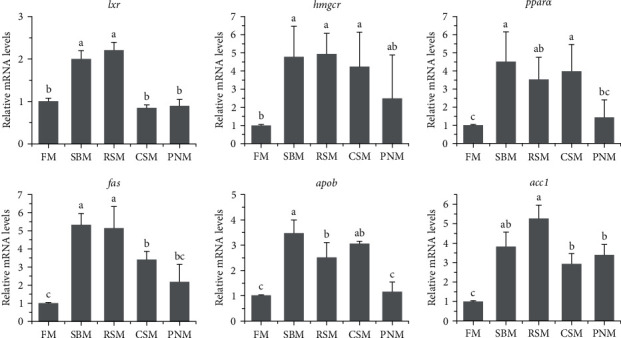
Expression levels of hepatic lipid metabolism-related genes in largemouth bass, *M. salmoides*, fed diets with different plant protein sources. The columns denoted by different letters are significantly different (*P* < 0.05, *n* = 3).

The expression levels of lipid metabolism-related genes, including *lxr*, *hmgcr*, *pparα*, *fas*, *apob*, and *acc1*, in the liver are shown in Figure 8. Statistical analysis showed that the mRNA levels of *hmgcr*, *pparα*, *fas*, *apob*, and *acc1* in the livers of fish fed the SBM, RSM, and CSM diets were significantly higher than those in the livers of fish fed the FM diet (*P* < 0.05). Most of the above genes in the livers of fish fed the PNM diet were also upregulated; however, only *acc1* was significantly different from that of fish fed the FM diet (*P* < 0.05). The expression levels of *lxr* in the liver of fish fed the SBM and RSM diets were significantly higher than those in fish fed the other diets (*P* < 0.05).

## 4. Discussion

### 4.1. Growth Performance

To select economical and accessible substitutes for FM, many scholars have carried out comparative studies on the utilization of different PPS [32–35]. Decreased SGR was observed in juvenile red claw crayfish, *Cherax quadricarinatus*, fed a diet with half of the FM (i.e., 24.5% of FM) isonitrogenously replaced by CSM, SBM, RSM, and PNM (PPS was included from 32% to 41%); however, a significant difference was observed only in PNM [35]. In hybrid tilapia (*Oreochromis niloticus* × *Oreochromis aureus*) fed isonitrogenous and isoenergetic diets, the SGR, FER, and PER in fish fed SBM, CSM, and PNM diets, respectively, were higher than those in fish fed the RSM diet (*P* < 0.05), in which the PPS ranged from 33% to 50% [36]. Prawns fed the SBM diet and CSM had higher WG and lower FCR than those fed the RSM diet and PNM diet (*P* < 0.05); the PPS ranged from 16.5% to 22% [18]. The results of this study showed that the growth performance of fish fed any of the diets containing 25% PPS was significantly lower than that of fish fed the FM diet (*P* < 0.05); among the four PPS groups, fish fed the RSM diet had the worst growth performance. The results of this study are consistent with previous reports, suggesting that high inclusion of PPS in the diet would reduce fish growth performance and feed utilization.

Among the four diets containing PPS, the RSM diet had the lowest ADCs of dry matter, protein, and lipids, and the PNM diet had the highest ADCs. These findings are in agreement with those of the PER and SGR, suggesting that the low ADC of nutrients is one of the reasons for the poor growth performance of fish fed a diet containing PPS. In addition, the decrease in FI (Table 5) should also contribute to the low SGR of fish fed diets containing PPS.

### 4.2. Bile Acid

The TBA content in the liver of fish fed any of the diets containing PPS was higher than that of fish fed the FM diet, although a significant difference was observed only in the RSM group (Figure 4). *cyp7a1*, *cyp8b1*, and *cyp27a1* encode rate-limiting enzymes in the BA synthesis pathway [37, 38], and the results of this study showed that the expression levels of the above two or all three genes in fish fed SBM, RSM, and CSM diets were significantly upregulated together (*P* < 0.05, Figure 7). In addition, cholestasis was observed in the liver tissue sections of fish fed the RSM diet (Figure 2(c1)). These results suggest that PPS induced the hypersynthesis of BA. Upregulation of BA synthesis was also observed by previous studies when PPS was included in the fish diets [27, 39, 40]. However, additional studies have suggested that PPS causes a decrease in BA levels in blood or bile [41–43]. The opposite effects may be attributed to the rhythm of BA synthesis. Murashita et al. [44] reported that the expression levels of BA synthesis genes in the liver of fish fed the SBM-based diets were higher than that of fish fed the FM diet in a short-term feeding trial (<24 hr), while in the long-term feeding trial (10 weeks), they had significantly lower expression levels.

In addition to the TBA content, the BA profiles in the liver also varied significantly among groups, and six types of BA, i.e., TDCA, apoCA, alloLCA, NorCA, THCA, and *λ*MCA could be used to distinguish diets very well, with the discriminatory accuracy reaching 100% (Figure 6). These findings suggest that the BA profile was sensitive to PPS. All the BAs that were screened into the discriminative model were secondary BAs, which are produced by intestinal microflora. It was therefore speculated that a great differentiation in the structure and/or function of intestinal microflora was induced by different PPS, which has often been observed [40, 45].

### 4.3. Histology of the Liver and Gut

There was a decreasing trend in the size of the liver of fish fed any of the diets containing PPS (Figure 1), and the HSI was significantly lower than that of fish fed the FM diet (*P* < 0.05, Table 9). Decreased HSI has been reported in tiger puffer, *Takifugu rubripes* [46], redlip mullet, *Liza hematocheila* [1], and largemouth bass [47] when the diet contained high level of PPS. The reason may lie in the high level of TBA in the liver. BA has cytotoxicity and can directly induce tissue necrosis [48, 49], which was observed in liver sections of fish fed the RSM (Figure 2) diet and PNM diet in this study. BA can also cause oxidative stress and mitochondrial dysfunction and then lead to the production of proinflammatory cytokines [50–52]. Proinflammatory cytokines further cause liver cell apoptosis [52, 53]. Nuclear factor (NF-*κβ*1), tumor necrosis factor-*α* (TNF-*α*), and interleukin-1*β* (IL-1*β*) are typical proinflammatory cytokines, and the expression of *tnf-α*, *il-1β*, and *nf-κβ1* in fish fed a diet containing PPS was upregulated (Figure 7). It was therefore speculated that the tissue necrosis, inflammatory reaction, and cell apoptosis induced by high concentrations of TBA may be important reasons for the decrease in liver size and HSI in fish fed diets containing PPS. AST and ALT are released into the blood when the liver tissue is injured, which should be the reason for the increased plasma AST and ALT activity in fish fed a diet containing PPS (Table 8).

This study and existing reports [1, 46, 47, 54, 55] showed that replacing FM with PPS would cause a decrease in HSI; however, there were also studies showing liver enlargement or increases in HSI in such conditions [2, 56, 57]. In this study, the liver tissue sections of fish fed the RSM and PNM diets clearly showed that some hepatocytes were swollen and ballooned, while others were atrophic and necrotic. It was therefore speculated that HSI decreases when hepatocytes generally decrease or become necrotic. However, when most of the cells in the liver are swollen or ballooned, the liver may be enlarged, and the HSI may increase.

White liver generally indicates steatosis [58, 59], and *apob* is highly expressed in fatty liver [60]. In this study, the mRNA level of *apob* was higher in fish fed the diet containing PPS than in those fed the FM diet (Figure 8). The color (Figure 1) and histology of the liver (Figure 2) and the liver lipid content (Figure 4) all suggested that hepatic steatosis occurred in fish fed the diet containing PPS, with the most serious steatosis in fish fed the RSM diet. *acc1* and *fas* encode acetyl-CoA carboxylase 1 and fatty acid synthesis, respectively, which are the key enzymes catalyzing the *de novo* synthesis of fatty acids [61]. FAS synthetic products can be used as endogenous activators of PPAR*α* [62], which participates in the regulation of lipid production [62] and homeostasis [63]. In this study, the expression levels of *acc1* in fish fed any of the diets containing PPS were higher than those in fish fed FM (*P* < 0.05), and the expression levels of *pparα* and *fas* in fish fed the SBM, RSM, and CSM diets was also higher than those in fish fed the FM diet (*P* < 0.05). These findings indicate that the *de novo* synthesis of lipids in the livers of fish fed diets containing PPS was enhanced and that the homeostasis of fat metabolism was disrupted. These might be the reasons for the increase in liver lipid content. Medical studies have also shown that hepatic steatosis is often accompanied by increased *de novo* synthesis of fatty acids [64, 65].

All factors causing chronic injury of liver tissue can activate a variety of cells and pathways and then cause a repair reaction. This repair reaction will cause the deposition of extracellular matrix (ECM, which is a fibrosis component) and then cause liver fibrosis [51]. At higher than physiological concentrations, BA is an important cause of chronic tissue damage, which can also lead to liver fibrosis [48]. In addition, upregulated expression of *tnf-α*, *il-1β*, and *nf-κβ1* aggravates fibrosis [50]. All these reasons contributed to the hepatic fibrosis observed in fish fed any of the diets containing PPS. According to the collagen content in the liver, the liver injury and repair reactions in fish fed the RSM diet were the strongest.

Studies have shown that noninfective enteritis could be induced by SBM in salmon, which was mainly manifested by the widening of the submucosa and lamina propria and lymphocyte infiltration [3, 66]. Enteritis induced by SBM can be repaired after fasting for 2 days [66]. In this study, no symptoms mentioned above were noticed, and only more exfoliated epithelial cells were observed in the intestinal lumen of fish fed a diet containing PPS than in that of fish fed an FM diet, which might indicate an enhanced capacity for repairing the intestine.

### 4.4. Potential Pathogenic Mechanisms

The results of this study indicated that the agent caused a decrease in ADCs and liver damage existed in all four selected PPSs, and DF was suspected. It is well-known that DF in the diet decreases the nutrient ADC [67]. More importantly, DF can bind BA [27, 68, 69], and BAs are also signaling molecules [70] and endogenous ligands for farnesoid X receptor (FXR) [71], which negatively regulate BA synthesis [71]. In other words, when part of the BAs in the intestine is trapped by DF, FXR activity is inhibited, resulting in excessive synthesis of BAs [14–16, 72]. This explains the increased TBA content in the liver and the change in BA profiles. CDCA functions as the most effective FXR agonist, while disruption of FXR results in generalized inflammation [73]. The results of this study showed that the CDCA content in the liver of fish fed a diet containing any type of PPS was lower than that of fish fed the FM diet, with the fish fed the RSM diet being the lowest (*P* < 0.05, Figure 5). These results were in agreement with the degree of liver injury. Insufficient FXR activity may further enhance the expression of *fxr*, as observed in this study (Figure 7). In addition, the liver lipid and collagen contents were significantly correlated with DF as well as SDF in the diet. All these results support that DF might be an important pathogenic agent in PPS.

*α*/*β*MCAs and their glycine and taurine derivatives are FXR antagonists in mice [73]. In this study, however, no significant increase in the sum of MCAs was observed in fish fed the RSM diet, whose liver was seriously damaged, suggesting that liver injury could not be explained by the regulatory effect of MCA on FXR. BA-activated receptors include FXR, TGR5, SIPR, etc., and various BAs have different or even opposite activation effects on different receptors, balancing each other to maintain immune and metabolic homeostasis [73]. Therefore, the impact of DF contained in PPSs on BA homeostasis and the resulting physiological reactions in fish are worth further study.

Cholesterol is the precursors for the synthesis of BAs, and LXR*α* participates in regulating cholesterol efflux and maintaining the homeostasis of intracellular cholesterol [74]. HMG-CoA reductase (HMGCR) is the key enzyme for cholesterol synthesis [75]. The results of this study showed that the expression levels of *hmgcr* in the liver of fish fed the SBM diet, RSM diet, and CSM diet were higher than those fed the FM diet (*P* < 0.05), and the expression levels of *lxr* in fish fed the SBM and RSM diet were also significantly higher than those fed the FM diet (*P* < 0.05). These findings suggest that cholesterol synthesis and efflux were enhanced, which may be to meet the need of the hypersynthesis of BA. However, the plasma TC content of fish fed a diet containing PPS was lower than that of fish fed an FM diet, suggesting that the hypersynthesis of cholesterol was insufficient to compensate for the consumption in BA synthesis. Hypocholesterolemia is one of the important effects of DF [67, 72].

When the level of PPS in diet is low in the diet, DF might mainly exert positive effects, such as improving the health of the liver and intestine [76]. Under this condition, the imbalance of amino acids may be an important factor that limits PPS utilization. The amino acid balance of SBM is better than that of CSM, PNM, and RSM [77]; therefore, SBM showed better growth performance than the other PPS [18]. However, when the inclusion level of PPS was high, the antinutritional effect of DF was highlighted, especially when cholesterol was in shortage [39]. The effect of DFs on BA homeostasis is related not only to their content and viscosity but also to their fermentability [13, 69]. Fermentable DF could affect the composition of intestinal microorganisms as well as their metabolites, thus producing a stronger physiological impact than nonfermentable DF [14, 69]. The DF in soybean has strong fermentability, which can be demonstrated from traditional fermented soybean foods, such as soy sauce, soybean paste, lobster sauce, and sufu. When the supplementation level of SBM was high in the diet, the advantage in amino acid balance might be masked by the antinutritional effect of DF. This might be attributed to the lowest HSI in fish fed the SBM diet and the lack of advantages compared to CSM in fish growth (Table 5) [35, 36].

## 5. Conclusion

The inclusion of 25% of each SBM, RSM, CSM, and PNM in the diet exceeded the tolerance range of largemouth bass and caused liver damage, which might be mediated by BA. DF in PPS might be an important agent contributing to liver damage.

## Figures and Tables

**Table 1:** Approximate composition of the main protein ingredients used in this study (g/kg air-dry basis).

Composition	FM	SBM	RSM	CSM	PNM
Moisture	101	115	108	89	112
Protein	669	430	379	519	528
Lipid	88	17	17	14	15
Ash	169	68	75	64	59
Total dietary fiber	4	361	392	303	278
Soluble dietary fiber	2	53	49	33	31

**Table 2:** Formulation and approximate composition of the experimental diets.

	FM	SBM	RSM	CSM	PNM
Ingredients (g/kg air-dry basis)					
Fish meal	550	300	300	300	300
Soybean meal	—	250	—	—	—
Rapeseed meal	—	—	250	—	—
Cottonseed meal	—	—	—	250	—
Peanut meal	—	—	—	—	250
Casein	60	130	144	104	101
Fish oil	0	22	22	22	22
Soybean oil	40	36	36	36	36
Zeolite power	104	16	2	42	45
Squid paste	20	20	20	20	20
Dextrin	180	180	180	180	180
Monocalcium phosphate	15	15	15	15	15
Premix	30	30	30	30	30
Yttrium oxide	1	1	1	1	1
Total	1,000	1,000	1,000	1,000	1,000
Approximate compositions					
Moisture	112	113	115	118	114
Protein	423	424	418	424	427
Lipid	88	86	84	87	88
Ash	191	99	89	115	123
Total dietary fiber	1.2	10.1	10.8	8.8	7.9
Soluble dietary fiber	0.6	1.9	1.8	1.4	1.3

*Note*: Premix contains vitamins and minerals. The guarantee values are the same as in the previous literature [16].

**Table 3:** The condition for bile acid (BA) LC–QqQ–MS analysis and the detected type of BA.

No.	Compound	Q1	Q3	Declustering potential	Collision energy (eV)	Cell exit potential	Detected BA
1	TDCA	498	498	−67	−65	−29	+
2	HDCA	391.4	391.4	−177	−21	−6	+
3	GUDCA	448.3	74.2	−144	−74	−8	—
4	GHDCA	448.3	74.1	−83	−77	−4	—
5	DCA	391.3	391.3	−12	−14	−12	+
6	TCDCA	498.3	498.3	−40	−38	−10	+
7	THDCA	498.3	498.3	−39	−11	−15	+
8	GCDCA	448.4	74.1	−43	−67	−9	—
9	TCA	514.2	514.2	−12	−12	−15	+
10	GDCA	448.3	74.1	−109	−77	−5	—
11	TUDCA	498.4	498.4	−41	−22	−15	+
12	UDCA	391.4	391.4	−10	−14	−12	+
13	CDCA	391.4	391.4	−25	−30	−12	+
14	GCA	464.4	74.1	−20	−75	−10	—
15	CA	407.5	407.5	−28	−15	−8	+
16	GLCA-1	432.3	74	−140	−70	−9	+
	GLCA-2	432.3	388.4	−82	−45	−12	—
17	apoCA-1	389.3	371.4	−194	−41	−11	+
	apoCA-2	389.3	59.1	−56	−41	−9	—
18	7,12-diketoLCA	401.3	401.3	−225	−31	−8	+
19	TLCA	482.3	482.3	−50	−25	−14	+
20	alloLCA-1	375.2	75	−50	−21	−9	+
	alloLCA-2	375.2	375.2	−188	−17	−22	—
21	MDCA	391.4	391.4	−113	−38	−24	+
22	12-DHCA	405.2	405.2	−70	−39	−4	+
23	LCA	375.3	375.3	−68	−32	−6	+
24	T*β*MCA	514.2	514.2	−91	−21	−4	+
25	GDHCA	458.3	73.8	−202	−78	−11	—
26	*β*MCA	407.2	407.2	−184	−40	−8	+
27	*β*HDCA	391.3	391.3	−128	−15	−8	+
28	NorCA-1	393.3	275.3	−225	−55	−19	+
	NorCA-2	393.3	329.3	−242	−46	−19	—
29	*β*UDCA	391.4	391.4	−191	−21	−26	+
30	dehydroLCA	373.3	373.3	−138	−39	−3	+
31	isoLCA	375.3	375.3	−214	−19	−7	+
32	UCA-1	406.9	325.3	−135	−51	−33	+
	UCA-2	406.9	343.2	−90	−45	−33	—
33	ACA-1	407.3	233.1	−136	−57	−13	+
	ACA-2	407.3	361.4	−141	−44	−11	—
34	NorDCA	377.3	377.3	−184	−38	−9	—
35	3-DHCA	405.3	405.3	−116	−39	−4	—
36	6-ketoLCA-1	389.2	59.1	−57	−39	−10	+
	6-ketoLCA-2	389.2	371.3	−179	−39	−7	—
37	7-ketoDCA	405.1	405.1	−157	−40	−8	+
38	12-ketoLCA	389.4	389.4	−4	−29	−13	+
39	THCA	514.3	514.3	−29	−13	−30	+
40	isoDCA	391.3	391.3	−58	−14	−25	+
41	*λ*MCA	407.3	407.3	−5	−24	−25	+
	*λ*MCA	407.3	389.3	−15	−47	−20	—
42	aMCA	407.1	407.1	−17	−14	−23	+
43	*ω*MCA	407.4	407.4	−163	−17	−12	+
44	CA-D4	411.4	290	−200	−53	−17	—

*Note*: +means detected BA.

**Table 4:** Real-Time PCR primer sequences.

Primer names	(5ʹ‒3ʹ) forward primer	(3ʹ‒5ʹ) reverse primer	Reference
*β-actin*	ATCGCCGCACTGGTTGTTGAC	CCTGTTGGCTTTGGGGTTC	[24]
*cyp7a1*	CTGGGCTTCACAGGCTAACACC	TTCAGTGTGGGGTCGTTGGG	[25]
*cyp8b1*	TAGACAGCGGCAACCAGGAG	CCGTGCTTTTGTTTCATCCTATC	[25]
*cyp27a1*	ATGCCCGTGTCACTGTTG	GTGCGGCTTGGACTTCTC	[26]
*fxr*	AGAAATGGCAACAAGTCAA	CACGGTCCAGAGAGAGAAA	[26]
*nf-κβ1*	GCTGCTCGTTGTCGTGAATA	CCACTTCACCCCTGATGACT	[27]
*il-1β*	CGTGACTGACAGCAAAAAGAGG	GATGCCCAGAGCCACAGTTC	[28]
*tnf-α*	CTTCGTCTACAGCCAGGCATCG	TTTGGCACACCGACCTCACC	[25]
*il-10*	CGGCACAGAAATCCCAGAGC	CAGCAGGCTCACAAAATAAACATCT	[25]
*tgf-β1*	GCTCAAAGAGAGCGAGGATG	TCCTCTACCATTCGCAATCC	[25]
*lxr*	ACAGCCCAGATCACTTT	ATGACACCCTCACAGCA	[29]
*hmgcr*	GGTGGAGTGCTTAGTAATCGG	ACGCAGGGAAGAAAGTCAT	[26]
*pparα*	CCACCGCAATGGTCGATATG	TGCTGTTGATGGACTGGGAAA	[25]
*fas*	CAGCCCTTGACTCATTCCG	CGCAGACTACGACCCGACAG	[30]
*acc1*	ATCCCTCTTTGCCACTGTTG	GAGGTGATGTTGCTCGCATA	[31]
*apob*	AGGCTGGGTGTTGTTGATGG	GAGAGCTGAGGGATGTTCTTGTTTAG	[25]

*Note*: *β-actin*, reference gene; *cyp7a1*, encodes cholesterol 7 *α*-hydroxylase; *cyp8b1*, encodes sterol 12 *α*-hydroxylase; *cyp27a1*, encodes cholesterol 27*α* hydroxylase; *fxr*, encodes farnesol X receptor; *nf-κβ1*, encodes nuclear factor kappa *β* subunit 1; *il-1β*, encodes interleukin 1 complex; *tnf-α*, encodes tumor necrosis factor alpha; *il-10*, encodes interleukin 10; *tgf-β1*, encodes transforming growth factor-*β*1; *lxr*, encodes liver X receptor; *hmgcr*, encodes 3-hydroxy-3-methylglutaryl-coenzyme A reductase; *pparα*, encodes peroxisome proliferator-activated receptor alpha; *fas*, encodes fatty acid synthase; *acc1*, encodes acetyl CoA carboxylase; and *apob*, encodes apolipoprotein.

**Table 5:** Effects of four types of plant protein sources on the growth and feed utilization of the largemouth bass, *M. salmoides*.

Diets	FM	SBM	RSM	CSM	PNM
Initial body weight (g/fish)	12.51 ± 0.06	12.49 ± 0.03	12.50 ± 0.25	12.47 ± 0.07	12.51 ± 0.04
Final body weight (g/fish)	54.94 ± 1.69^a^	48.64 ± 0.73^bc^	46.30 ± 1.14^c^	48.81 ± 2.44^bc^	50.09 ± 1.20^b^
Weight gain (%)	339.43 ± 11.72^a^	289.44 ± 5.09^bc^	270.58 ± 3.24^c^	291.59 ± 21.68^bc^	300.47 ± 8.58^b^
Specific growth rate (%/day)	2.64 ± 0.05^a^	2.43 ± 0.02^bc^	2.34 ± 0.02^c^	2.44 ± 0.10^bc^	2.48 ± 0.04^b^
Feed intake (g/fish/day)	0.83 ± 0.02^a^	0.81 ± 0.03^b^	0.80 ± 0.02^b^	0.82 ± 0.02^ab^	0.82 ± 0.01^ab^
Feed conversion ratio	1.09 ± 0.04^b^	1.22 ± 0.07^ab^	1.29 ± 0.09^a^	1.24 ± 0.05^a^	1.19 ± 0.11^ab^
Protein efficiency ratio	2.12 ± 0.10^a^	1.87 ± 0.09^b^	1.83 ± 0.02^b^	1.88 ± 0.09^b^	1.96 ± 0.09^b^
Survival (%)	100 ± 0.00	96.67 ± 2.89	100 ± 0.00	96.67 ± 2.89	96.67 ± 5.77

*Note*: Values in the same row with different letters are significantly different (*P* < 0.05, *n* = 3).

**Table 6:** Effects of four types of plant protein sources on the whole body composition of largemouth bass, *M. salmoides*.

Diets (g/kg)	FM	SBM	RSM	CSM	PNM
Moisture	694.3 ± 8.7	697.0 ± 4.6	700.3 ± 4.7	695.6 ± 2.3	693.3 ± 5.6
Protein	164.8 ± 3.2	163.4 ± 1.1	161.1 ± 2.3	162.5 ± 0.3	158.2 ± 7.0
Lipid	68.7 ± 2.9	67.0 ± 3.6	72.8 ± 3.0	70.3 ± 6.0	70.2 ± 3.6
Ash	39.9 ± 3.5	38.3 ± 2.4	38.3 ± 2.5	40.0 ± 1.2	38.9 ± 2.1

**Table 7:** Effects of four types of plant protein sources on the apparent digestibility coefficients of dry matter, protein, and lipids for largemouth bass, *M. salmoides*.

Diets (%)	FM	SBM	RSM	CSM	PNM
Dry matter	79.6 ± 0.9^a^	78.4 ± 1.6^ab^	75.3 ± 2.3^b^	76.9 ± 1.5^ab^	78.8 ± 1.9^a^
Protein	91.4 ± 0.8^a^	89.3 ± 1.4^abc^	87.5 ± 1.9^c^	88.8 ± 1.2^bc^	90.6 ± 0.9^ab^
Lipid	93.4 ± 1.0	91.1 ± 0.8	90.3 ± 2.3	91.3 ± 1.1	92.3 ± 0.5

*Note*: Values in the same row with different letters are significantly different (*P* < 0.05, *n* = 3).

**Table 8:** Effects of four types of plant protein sources on the plasma biochemistry of largemouth bass, *M. salmoides*.

Diets	FM	SBM	RSM	CSM	PNM
Aspartate aminotransferase (U/l)	28.3 ± 2.6^d^	51.3 ± 1.5^b^	64.7 ± 2.3^a^	34.7 ± 4.5^c^	66.0 ± 1.7^a^
Alanine aminotransferase (U/l)	7.3 ± 0.6^b^	7.7 ± 0.6^b^	13.0 ± 1.0^a^	8.7 ± 1.5^b^	8.0 ± 1.0^b^
Alkaline phosphatase (U/l)	113.7 ± 6.7^b^	124.0 ± 3.5^b^	169.0 ± 1.0^a^	113.7 ± 5.0^b^	167.3 ± 2.5^a^
Total cholesterol (mmol/l)	9.7 ± 0.5^a^	8.2 ± 0.2^bc^	6.7 ± 0.3^c^	7.2 ± 1.0^bc^	8.6 ± 0.5^b^
Triglyceride (mmol/l)	7.0 ± 0.2^d^	6.8 ± 0.4^d^	9.6 ± 0.1^a^	7.5 ± 0.4^c^	8.6 ± 0.4^b^

*Note*: Values in the same row with different letters are significantly different (*P* < 0.05, *n* = 3).

**Table 9:** Effects of four types of plant protein sources on the organ index of largemouth bass, *M. salmoides*.

Diets	FM	SBM	RSM	CSM	PNM
Viscerosomatic index (VSI, %)	10.89 ± 0.40^a^	8.09 ± 0.34^d^	8.99 ± 0.19^c^	9.48 ± 0.08^bc^	10.01 ± 0.50^b^
Hepatosomatic index (HSI, %)	4.05 ± 0.24^a^	2.59 ± 0.32^c^	2.72 ± 0.34^bc^	2.97 ± 0.33^bc^	3.25 ± 0.31^b^
Gallbladder somatic index (GBSI, %)	0.15 ± 0.01^b^	0.17 ± 0.01^ab^	0.19 ± 0.03^a^	0.19 ± 0.02^a^	0.17 ± 0.02^ab^

*Note*: Values in the same row with different letters are significantly different (*P* < 0.05, *n* = 3).

## Data Availability

The data that support the findings of this study are available from the corresponding author upon reasonable request.
